# Chloride Improves Nitrate Utilization and NUE in Plants

**DOI:** 10.3389/fpls.2020.00442

**Published:** 2020-05-26

**Authors:** Miguel A. Rosales, Juan D. Franco-Navarro, Procopio Peinado-Torrubia, Pablo Díaz-Rueda, Rosario Álvarez, José M. Colmenero-Flores

**Affiliations:** ^1^Grupo Regulación Iónica e Hídrica en Plantas, Instituto de Recursos Naturales y Agrobiología, Consejo Superior de Investigaciones Científicas (CSIC), Seville, Spain; ^2^Laboratorio Interdepartamental de Ecofisiología Molecular de Plantas, Instituto de Recursos Naturales y Agrobiología, Consejo Superior de Investigaciones Científicas (CSIC), Seville, Spain; ^3^BioScripts – Centro de Investigación y Desarrollo de Recursos Científicos, Seville, Spain; ^4^Departamento de Biología Vegetal y Ecología, Facultad de Biología, Universidad de Sevilla, Seville, Spain

**Keywords:** chloride, nitrate, nitrogen use efficiency, crop yield, fertilizer, tobacco, leafy vegetables, nutritional quality

## Abstract

Chloride (Cl^–^) has traditionally been considered harmful to agriculture because of its toxic effects in saline soils and its antagonistic interaction with nitrate (NO_3_^–^), which impairs NO_3_^–^ nutrition. It has been largely believed that Cl^–^ antagonizes NO_3_^–^ uptake and accumulation in higher plants, reducing crop yield. However, we have recently uncovered that Cl^–^ has new beneficial macronutrient, functions that improve plant growth, tissue water balance, plant water relations, photosynthetic performance, and water-use efficiency. The increased plant biomass indicates in turn that Cl^–^ may also improve nitrogen use efficiency (NUE). Considering that N availability is a bottleneck for the plant growth, the excessive NO_3_^–^ fertilization frequently used in agriculture becomes a major environmental concern worldwide, causing excessive leaf NO_3_^–^ accumulation in crops like vegetables and, consequently, a potential risk to human health. New farming practices aimed to enhance plant NUE by reducing NO_3_^–^ fertilization should promote a healthier and more sustainable agriculture. Given the strong interaction between Cl^–^ and NO_3_^–^ homeostasis in plants, we have verified if indeed Cl^–^ affects NO_3_^–^ accumulation and NUE in plants. For the first time to our knowledge, we provide a direct demonstration which shows that Cl^–^, contrary to impairing of NO_3_^–^ nutrition, facilitates NO_3_^–^ utilization and improves NUE in plants. This is largely due to Cl^–^ improvement of the N–NO_3_^–^ utilization efficiency (NU_T_E), having little or moderate effect on N–NO_3_^–^ uptake efficiency (NU_P_E) when NO_3_^–^ is used as the sole N source. Clear positive correlations between leaf Cl^–^ content vs. NUE/NU_T_E or plant growth have been established at both intra- and interspecies levels. Optimal NO_3_^–^ vs. Cl^–^ ratios become a useful tool to increase crop yield and quality, agricultural sustainability and reducing the negative ecological impact of NO_3_^–^ on the environment and on human health.

## Introduction

Nitrogen (N) is the main limiting nutrient for land plants and, therefore, has been classified as an essential macronutrient. Nitrate (NO_3_^–^) represents the major N source and a signal molecule involved in the control of many physiological and developmental processes, strongly improving crop yield (Frink et al., 1999; Wang et al., 2012; Krapp et al., 2014; Guan, 2017). The decisive role of N in crop yield has led to excessive use of NO_3_^–^ in agriculture over decades generating serious environmental problems like water pollution, which is harmful to people and nature (Nitrates Directive, 1991; Kant et al., 2011). In addition, when the application rate of NO_3_^–^ exceeds the plant growth needs, overaccumulation of NO_3_^–^ in leaves reduces the nutritional quality of crops (Prasad and Chetty, 2008; Xing et al., 2019). Many large-leaved plants such as beets, cabbage, celery, lettuce, or spinach tend to store huge amounts of NO_3_^–^ (MAFF, 1998), posing a serious risk to human health. When ingested, NO_3_^–^ is rapidly converted to nitrite and N-nitrous compounds as nitrosamines or nitric oxide causing *methemoglobinemia* or “blue baby syndrome” in infants and gastric cancer among other pathological disorders (Comly, 1945; Santamaria et al., 1999; Mensinga et al., 2003).

Considering that the growing world population is predicted to reach 9.8 billion in 2050, global efforts are being made to increase food resources improving crop or agronomic practices (Tilman et al., 2002; Godfray et al., 2010). Since only 30–40% of the N applied to soil is used by plants, a greater N use efficiency (NUE) could improve the yield and quality of crops, reducing economic costs as well as decreasing environmental degradation (Baligar et al., 2001). NUE can be defined as the vegetative or reproductive biomass yield per unit of N available in the soil (Moll et al., 1982; Woodend and Glass, 1993; Ríos et al., 2010). This concept has many variants that can be split into two main elements: (i) N uptake efficiency (NU_P_E), defined as the capacity of plant roots to take N from soil, and (ii) N utilization efficiency (NU_T_E), defined as the fraction of plant-acquired N to be converted to total biomass or grain yield (Xu et al., 2012). Both are considered important traits in agriculture to reduce the abusive use of N fertilizers or when N availability constrains plant growth, with substantial benefits for farmers and to the environment (Baligar et al., 2001; Han et al., 2016). Crops with higher NUE promote greater yields under limited N in soil, or require lower N to produce the same yield as those with lower NUE capacity (Ruiz et al., 2006; Kant et al., 2011; Rubio-Wilhelmi et al., 2012). Therefore, when NUE is increased, both crop-production costs and the harmful input of NO_3_^–^ into ecosystems are reduced.

Traditionally, chloride (Cl^–^) has been considered an essential micronutrient for plants (White and Broadley, 2001; Broadley et al., 2012). But recently, Cl^–^ has been uncovered as beneficial when accumulated to macronutrient levels in plant tissues (Franco-Navarro et al., 2016; Raven, 2017; Wege et al., 2017; Colmenero-Flores et al., 2019), with new biological functions that improve tissue water balance, whole-plant water relations, photosynthesis performance, and water-use efficiency (Franco-Navarro et al., 2016, 2019; Nieves-Cordones et al., 2019). Chloride represents the dominant inorganic anion in the vacuole, with leaf contents that can be similar to those of the macronutrient K^+^, promoting cell osmoregulation, turgor-driven processes, leaf cell elongation, and a reduction in stomatal conductance (*g*_s_; Franco-Navarro et al., 2016). In addition, Cl^–^ specifically increases mesophyll diffusion conductance to CO_2_ (*g*_m_) as a consequence of the greater surface area of chloroplasts exposed to the intercellular airspace of mesophyll cells, which in turn points towards Cl^–^ playing a role in chloroplast performance (Franco-Navarro et al., 2019). Thus, Cl^–^ specifically reduces *g*_s_ and water loss through transpiration without affecting the photosynthetic capacity due to *g*_m_ stimulation, resulting in overall higher water-use efficiency (Franco-Navarro et al., 2016, 2019; Maron, 2019). Nitrate and Cl^–^ are the most abundant inorganic anions, having similar physical and osmoregulatory properties and sharing transport mechanisms (Colmenero-Flores et al., 2019). This is probably the reason why NO_3_^–^ and Cl^–^ show strong dynamic interactions in plants (Wege et al., 2017), a phenomenon that has been described as a competitive interaction between these two monovalent anions. Different studies have reported a negative effect of Cl^–^ on root NO_3_^–^ uptake and accumulation (Siddiqi et al., 1990; Cerezo et al., 1997; Xu et al., 2000). For this reason and because of the toxicity generated by excessive Cl^–^ accumulation in sensitive crops under salt–stress conditions (Li et al., 2017; Geilfus, 2018), Cl^–^ has been considered detrimental to agriculture. Overall, Cl^–^ is believed to reduce NUE by limiting NO_3_^–^ uptake and accumulation in plant tissues, reducing in turn its availability for plant metabolism (Xu et al., 2000; Anjana and Iqbal, 2007; Wege et al., 2017). However, Cl^–^ is a non-metabolized anion whose vacuolar accumulation requires a lower energy cost than the accumulation of NO_3_^–^ (Wege et al., 2017). Thus, considering the close interactions between these two anions, it has been hypothesized that preferential Cl^–^ compartmentalization may reduce vacuolar NO_3_^–^ storage in leaves (Flowers, 1988), allowing higher NO_3_^–^ availability for plant metabolism and, consequently, promoting more efficient use of this N source, meaning higher NUE (Colmenero-Flores et al., 2019). Therefore, the goal of this study was to verify whether Cl^–^ reduces leaf NO_3_^–^ accumulation while promoting more efficient use of N–NO_3_^–^. In order to prove this, different plant species with contrasting Cl^–^-accumulating abilities have been used in this work: three leafy herbaceous species with strong Cl^–^-including capacity (chard, spinach, and lettuce), two herbaceous Cl^–^-including Solanaceae species (tobacco and tomato), and two Cl^–^-excluding woody species (olive and the salt-tolerant citrus rootstock Cleopatra mandarin). To directly ascertain the effect of Cl^–^ on NO_3_^–^ nutrition, plant growth and different NUE parameters have been quantified, using NO_3_^–^ as the sole N source.

## Materials and Methods

### Plant Species and Nutritional Treatments

Tobacco (*Nicotiana tabacum* L. var. habana) plants were grown under experimental greenhouse conditions at 25 ± 3°C/17 ± 2°C (day/night), relative humidity of 60 ± 10% (EL-1-USB Data-logger, Lascar Electronics Inc., Erie, PA, United States), a 14 h/10 h photoperiod with a photosynthetic photon flux density [average photosynthetically active radiation (PAR)] of 300–350 μmol m^–2^ s^–1^ (quantum sensor, LI-6400; Li-COR, Lincoln, NE, United States), and a luminous emittance of 9,000–10,000 lx (Digital Lux Meter, LX1010B; Carson Electronics, Valemount, Canada). Seeds were sown in flat trays (cell size, 4 cm × 4 cm × 10 cm) containing peat previously washed with the corresponding nutrient solutions. After 2 days of vernalization in a cold chamber (4°C), seedbeds were transferred to a greenhouse. 21 days after sowing (DAS), seedlings were transplanted to 7.5 L pots (with a pot size of 20 cm × 17 cm × 25 cm) that contained a mix of perlite/vermiculite (4:6). Plants were watered with a basal nutrient solution supplemented with three salt solutions containing the same cationic balance: 5 mM Cl^–^-based treatment (CL; with 5.075 mM Cl^–^ and 5.25 mM NO_3_^–^), 5 mM NO_3_^–^-based treatment (N; with 75 μM Cl^–^ and 10.25 mM NO_3_^–^) and sulfate + phosphate (SO_4_^2–^ + PO_4_^3–^)-based treatment (SP; with 75 μM Cl^–^ and 5.25 mM NO_3_^–^). The composition of the basal solution (BS) was as follows: 1.25 mM KNO_3_, 0.725 mM KH_2_PO_4_, 0.073 mM K_2_HPO_4_, 2 mM Ca(NO_3_)_2_, 1 mM MgSO_4_, 0.1 mM FeNa–ethylenediaminetetraacetic acid (EDTA), 0.1 mM H_3_BO_3_, 0.1 mM MnSO_4_, 29 μM ZnSO_4_, 0.1 μM CuSO_4_, 1 μM Na_2_MoO_4_, and 5 μM KI. A detailed description of the nutritional treatments is given in the Supplementary Table S1. Considering that 50 μM Cl^–^ was reported to ensure Cl^–^ micronutrient requirements in different plant species (Johnson et al., 1957), 75 μM Cl^–^ (added as 11 μM CoCl_2_ and 53 μM KCl, including water traces) was present in the basal nutrient solution to fulfill micronutrient Cl^–^ functions in low Cl^–^ treatments (Franco-Navarro et al., 2016, 2019). In these previous works, we showed that the SP supplement did not modify the parameters analyzed with respect to the baseline treatment (BS). For this reason, and because the SP treatment only modifies the anionic content with respect to the CL treatment (while the BS solution differs in both anionic and cationic content), the BS treatment was not included in this work. Furthermore, previous experiments showed no significant differences in NUE parameters between BS and SP treatments (results not shown). A second set of experiments with increasing concentrations of anions was used in CL treatments: 0 mM Cl^–^ (basal solution containing 0.075 mM Cl^–^), 0.151 mM Cl^–^, 0.301 mM Cl^–^, 1.075 mM Cl^–^, 2.575 mM Cl^–^, and 5.075 mM Cl^–^. As a control condition, equivalent SP treatments were used to ensure similar cationic balance as in the different CL treatments (Supplementary Table S1). All experimental solutions were adjusted to pH 5.7 with KOH. Pots were irrigated up to field capacity (3.5 mL g^–1^ substrate) along with the experiments. Tobacco plants were harvested at 64 DAS, and different plant tissues were preserved for subsequent analyses.

To find out the ratio of Cl^–^ vs. NO_3_^–^ that promotes more efficient use of N, tobacco plants were subjected to varying ratios of Cl^–^, NO_3_^–^, and SO_4_^2–^ + PO_4_^3–^ as follows (Supplementary Table S2): (i) constant 8 mM NO_3_^–^ combined with increasing Cl^–^ concentrations and decreasing SO_4_^2–^ + PO_4_^3–^ concentrations (mM; NO_3_^–^/SO_4_^2–^ + PO_4_^3–^: 0.075:8, 0.575:7.5, 2.075:6, 4.075:4, and 6.075:2) and (ii) constant 6.075 mM Cl^–^ combined with increasing SO_4_^2–^ + PO_4_^3–^ concentrations and decreasing NO_3_^–^ concentrations (mM; Cl^–^/SO_4_^2–^ + PO_4_^3–^: 6:4, 4:6). The minimum content of Cl^–^ was maintained at 75 μM to ensure the minimal micronutrient requirement (Franco-Navarro et al., 2016), which was estimated up to 50 μM in the nutrient solution as reported in Johnson et al. (1957) and Whitehead (1985), and salt combinations contained the same cationic balance.

SP and CL treatments (5 mM) were applied at 21 DAS under similar experimental conditions (as described above) in: (i) woody species like olive (*Olea europaea* L. ssp. *europaea* var. *sylvestris* Brot.) and the citrus rootstock Cleopatra mandarin (*Citrus reshni* Hort. ex Tan.); and (ii) herbaceous species like cherry tomato (*Solanum lycopersicum* L. cv *zarina*), Taglio chard (*Beta vulgaris* L. ssp. *vulgaris* convar. *cicla* var. *flavescens* Dc.), America spinach (*Spinacia oleracea* L. var. *america*), and lettuce romaine (*Lactuca sativa* ssp. *longifolia* Lam.). For olive plants, *in vitro* germination of zygotic embryos was required. Seeds were sterilized and germinated under sterile conditions in tubes containing 10 mL of olive culture medium (Rugini, 1984) supplemented with 1 mg L^–1^ zeatin, 20 g L^–1^ mannitol, and 6 g L^–1^ agar. Medium pH was adjusted to 5.7 before autoclaving at 121°C for 20 min. After placing the embryos in the agar medium, they were incubated in the growth chamber for 60 days. Growing conditions were 23 ± 2°C, 16 h light/8 h dark photoperiod, and 70%/30% Red/Blue with a photosynthetic photon flux (PPF) of 34 μE. Seedlings were placed in rooting medium for 21 days before being acclimatized in pots for 21 days and then harvested at 200 DAS. The other plant species were harvested at different times as follows: at 67 DAS in tomato, 84 DAS in mandarin, 106 DAS in spinach, and 147 DAS in chard and lettuce.

Plant samples harvested in all experiments were dried in a forced-air oven at 75°C to obtain the dry weight (DW) and dry preserved for subsequent determinations. All experiments were performed in at least three independent trials.

### Nutrient Content and NUE Parameters

For the determination of nutrient content, fully photosynthetic and expanded mature leaves (non-senescent) were used. Oven-dried leaf tissue was ground into powder using a grinder, and the concentration of Cl^–^, NO_3_^–^, SO_4_^2–^, and PO_4_^3–^ was determined as previously reported in Franco-Navarro et al. (2016). NH_4_^+^ was determined from an aqueous extraction by using the colorimetric method described by Krom (1980), and was measured with the absorbance microplate reader “Omega SPECTROstar” (BMG LABTECH GmbH, Germany). Organic N was determined by the Kjeldahl method (Bradstreet, 1954). Total N content (TNC) was expressed as mg g^–1^ DW and represents the sum of organic N, NH_4_^+^, and NO_3_^–^ (Ríos et al., 2010). Total N accumulation (TNA) was calculated as the result of TNC divided by total DW as described in Sorgona et al. (2006), and results were expressed as mg of N. NUE is commonly defined as vegetative yield per unit of N available to the crop (g DW g^–1^ N; Moll et al., 1982; Woodend and Glass, 1993; Rubio-Wilhelmi et al., 2012) and can be subdivided into two types: (i) N utilization efficiency (NU_T_E) calculated as total DW divided by TNC (g^2^ DW mg^–1^ N; Siddiqi and Glass, 1981) and (ii) N uptake efficiency (NU_P_E) calculated as TNA divided by root DW (mg N g^–1^ root DW; Elliott and Læuchli, 1985).

### Statistical Analysis

Statistical analysis was performed using the STATGRAPHICS Centurion XVI software (StatPoint Technologies, Warrenton, VA, United States). The Shapiro–Wilk (*W*) test was used to verify the normality of the datasets. Both one-way analysis of variance (ANOVA) and multivariate analysis of variance (MANOVA) were done to determine significant differences between groups of samples, and levels of significance were described by asterisks: *P* ≤ 0.05 (^∗^), *P* ≤ 0.01 (^∗∗^), and *P* ≤ 0.001 (^∗∗∗^). No significant (NS) differences were indicated when *P* > 0.05. Multiple comparisons of means were determined by the Tukey’s honestly significant difference (HSD) and multiple range test (MRT) tests included in the afore-mentioned software. Correlations between NUE parameters and Cl^–^ concentrations were calculated through Pearson’s product-moment correlation coefficient (*r*^2^). Values represent the mean of at least five tobacco plants in each treatment, which were reproducible in at least two independent experiments.

## Results

### Effect of Cl^–^ on Leaf Ion Content, Growth, and NUE Parameters in Tobacco Plants

**FIGURE 1:**
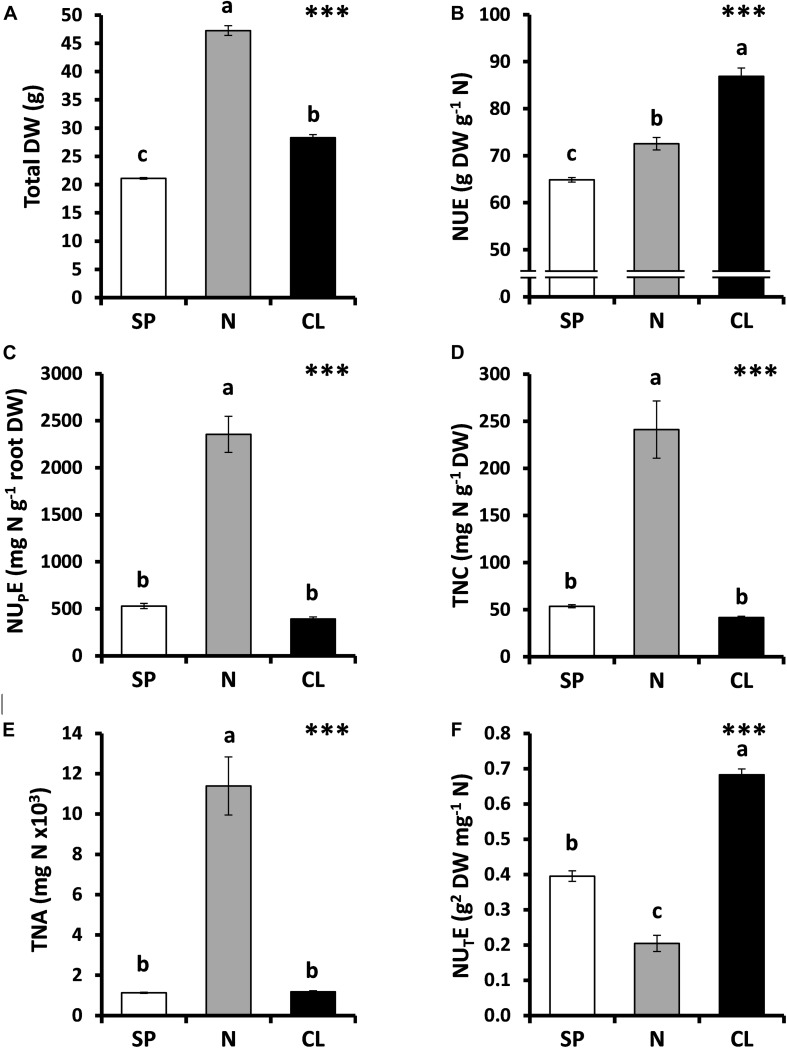
Effect of Cl^–^ nutrition on tobacco biomass and nitrogen use efficiency (NUE) parameters. Treatments consisted of the application of the basal nutrient solution supplemented with 5 mM Cl^–^ (CL), 5 mM NO_3_^–^ (N), or the SO_4_^2–^ + PO_4_^3–^ (SP) salt mixture, containing the same cationic balance in all treatments. **(A)** Total dry weight (DW). **(B)** NUE. **(C)** Nitrogen-uptake efficiency (NU_P_E). **(D)** Total nitrogen content (TNC). **(E)** Total nitrogen assimilated (TNA). **(F)** Nitrogen-utilization efficiency (NU_T_E). Mean values ± SE, *n* = 4-6. Levels of significance: ^∗∗∗^*P* ≤ 0.001; and “homogeneous group” statistics was calculated through ANOVA tests, where mean values with different letters are significantly different according toTukey’s test.

**FIGURE 2:**
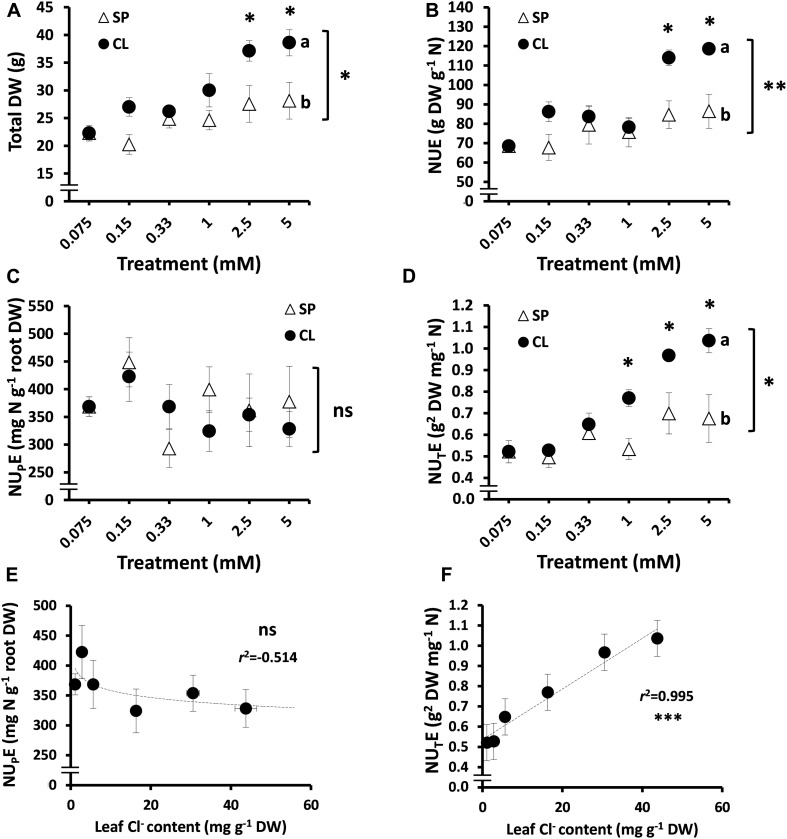
Effect of Cl^–^ nutrition on tobacco plant biomass and nitrogen use efficiency (NUE) parameters. Treatments consisted of increasing concentrations of Cl^–^ (CL) or SO_4_^2–^ + PO_4_^3–^ (SP) salts maintaining the same cationic balance. **(A)** Effect on total dry weight (DW). **(B)** NUE. **(C)** Nitrogen-uptake efficiency (NU_P_E). **(D)** Nitrogen-utilization efficiency (NU_T_E); **(E)** Pearson correlation (*r*^2^) between NU_P_E and leaf anion content in tobacco plants. **(F)** Pearson correlation (*r*^2^) between NU_T_E and leaf anion content in tobacco plants. Mean values ± SE, *n* = 4–6. Levels of significance: *P* > 0.05 (ns, not significant), ^∗^*P* ≤ 0.05, ^∗∗^*P* ≤ 0.01, and ^∗∗∗^*P* ≤ 0.001; and “homogeneous group” statistics was calculated through ANOVA and multivariate (MANOVA) tests, where mean values with different letters are significantly different according toTukey’s test. Correlations between NU_T_E or NU_P_E and leaf anion content were calculated through the Pearson’s product-moment correlation coefficient (*r*^2^).

The three nutritional treatments assayed (SP, N, and CL) showed leaf ionic contents consistent with the nutritional supplements applied (Supplementary Table S3). Thus, CL plants accumulated Cl^–^ at levels that are typical of a macronutrient such as K^+^ (55.1 mg Cl^–^ g^–1^ DW and 49.5 mg K^+^ g^–1^ DW, respectively). Leaf Cl^–^ content in CL plants was higher than the contents of NO_3_^–^ and SO_4_^2–^ + PO_4_^3–^ in N and SP plants, respectively (Supplementary Table S3). It is important to notice that the leaf Cl^–^ content in tobacco plants treated with low Cl^–^ levels (SP and N treatments) exceeded the critical deficiency threshold reported for Cl^–^ in non-halophytic plants (<0.2 mg g^–1^ shoot DW; Flowers, 1988; Xu et al., 2000; White and Broadley, 2001). Therefore, N and SP treatments satisfied plant Cl^–^ requirements as essential micronutrient, and no symptoms of Cl^–^ deficiency like wilting, chlorosis, bronzing, or necrosis were observed. As a demonstration of this fact, we noted that N plants, containing low Cl^–^ content, exhibited the highest plant growth (Franco-Navarro et al., 2016; Figure 1A). As previously observed, Cl^–^ supplementation stimulated plant growth (when compared to the SP treatment) (Figures 1A, 2A). Interestingly, the beneficial effect of Cl^–^ nutrition on plant dry biomass was only evident in response to treatments higher than 1 mM Cl^–^, within the macronutrient-content range (Figure 2A). Therefore, these results show that Cl^–^ stimulates plant growth when it is supplied at macronutrient levels and ruled out the occurrence of Cl^–^ deficiency in plants subjected to low Cl^–^ treatments (SP and N treatments).

In tobacco plants, the N treatment (10.25 mM NO_3_^–^) duplicated the NO_3_^–^ concentration in comparison with SP and CL treatments (5.25 mM NO_3_^–^), resulting in strong stimulation of plant growth (Supplementary Table S1 and Figure 1A) and confirming the well-known fact that N availability bottlenecks plant growth (Glass, 2003; Hawkesford et al., 2012; Wang et al., 2012; Krapp et al., 2014; Guan, 2017). However, the most efficient use of N occurred in CL plants, which showed the highest NUE values (Figure 1B) despite presenting the lowest NO_3_^–^ content (Supplementary Table S3). NUE defines the total biomass production per unit of N (NO_3_^–^) available in the soil (Moll et al., 1982). Two different components of NUE can be in turn distinguished: (i) how efficiently is this nutrient transported into the plant, defined by the N uptake efficiency (NU_P_E), and (ii) how efficiently the transported N is used by the plant, defined by the N utilization efficiency (NU_T_E), which takes into account the plant yield component (Siddiqi and Glass, 1981). As a result of the greater NO_3_^–^ availability, the N treatment resulted in a strong increase in NU_P_E (Figure 1C), giving rise to higher TNC (Figure 1D) and TNA (Figure 1E) in comparison to the SP and CL treatments. However, such high tissue content of N determined the lowest NU_T_E value in N plants (Figure 1E), which was 70% lower than that of CL plants. Interestingly, while both CL and SP treatments contained the same NO_3_^–^ concentration, the CL treatment determined 41% higher NU_T_E than the SP treatment.

To better define the interaction between Cl^–^ and NUE, the plant response to increasing Cl^–^ concentrations was compared to equivalent gradients of SO_4_^2–^ + PO_4_^3–^ concentrations. A clear positive response to Cl^–^ treatments was observed beyond 1 mM Cl^–^, significantly improving plant growth (Figure 2A) and NUE (Figure 2B) in comparison to SP treatments. These CL treatments determined leaf tissue contents of about 40–110 mM Cl^–^, confirming the beneficial effect of Cl^–^ at macronutrient levels. Interestingly, no significant differences were observed in the NU_P_E between the CL and SP treatments (both containing the same concentration of 5.25 mM NO_3_^–^; Figure 2C), whereas NU_T_E values were higher in CL plants subjected to treatments ≥ 1 mM Cl^–^ (Figure 2D). This confirmed that the NUE component improved by Cl^–^ is the utilization rather than the uptake efficiency of NO_3_^–^. Thus, a positive and statistically significant correlation between NU_T_E and leaf Cl^–^ content was confirmed (*r*^2^ = 0.99; Figure 2F), which could not be established with the NU_P_E (Figure 2E) in tobacco plants.

### Effect of Different Cl^–^/NO_3_^–^ and Cl^–^/SO_4_^2–^ + PO_4_^3–^ Ratios on Anion Content, Growth, and NUE Parameters of Tobacco Plants

**FIGURE 3:**
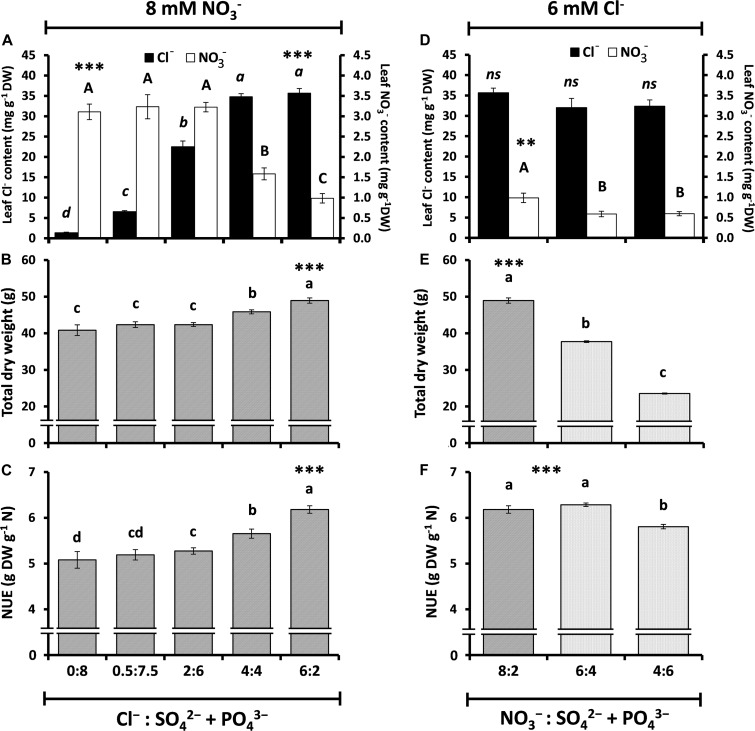
Effect of different ratios of Cl^–^ nutrition on anion content, plant growth, and nitrogen use efficiency (NUE) in tobacco plants. Treatments consisted of the application of: (**A–C**; ↑Cl^–^/↓SO_4_^2–^ + PO_4_^3–^) increasing concentrations of Cl^–^ (from 0.075 to 6 mM) and decreasing concentrations of SO_4_^2–^ + PO_4_^3–^ (from 8 to 2 mM) while keeping constant the concentration of NO_3_^–^ (8 mM); and (**D–F**; ↓NO_3_^–^/↑SO_4_^2–^ + PO_4_^3–^) decreasing concentrations of NO_3_^–^ (from 8 to 4 mM) and increasing concentrations of SO_4_^2–^ + PO_4_^3–^ (from 2 to 6 mM) while keeping constant the concentration of Cl^–^ (6 mM). **(A,D)** Effect on leaf anion contents (NO_3_^–^ and Cl^–^). **(B,E)** Effect on total dry weight (DW). **(C,F)** Effect on nitrogen-use efficiency (NUE). Mean values ± SE, *n* = 6. Levels of significance: *P* > 0.05 (ns, not significant differences); ^∗∗∗^*P* ≤ 0.001; and “homogeneous group” statistics was calculated through ANOVA tests, where mean values with different letters are significantly different according toTukey’s test.

To better understand whether Cl^–^ has a direct antagonistic effect on NO_3_^–^ nutrition, and therefore on plant performance, tobacco plants treated with the same NO_3_^–^ concentration (8 mM NO_3_^–^) were supplemented with growing Cl^–^ concentrations (0, 0.5, 2, 4, and 6 mM Cl^–^). To maintain a similar cationic and osmotic balance in all treatments, Cl^–^ salts were compensated with SO_4_^2–^ + PO_4_^3–^ salts according to the experimental design presented in Supplementary Table S2. Increasing Cl^–^ concentrations gave rise to increasing leaf Cl^–^ contents, which in turn produced significant reductions in NO_3_^–^ content in the 4 and 6 mM Cl^–^ treatments (53 and 71% reduction in NO_3_^–^ content, respectively; Figure 3A). Interestingly, these strong reductions in leaf NO_3_^–^ content did not result in a worsening of plant performance, and contrary to what is traditionally believed, Cl^–^ treatments significantly increased plant biomass (Figure 3B) and NUE (Figure 3C). The results clearly suggest that a reduction in NO_3_^–^ content by Cl^–^ application is not due to a reduction in NO_3_^–^ availability within the plant but to a greater NO_3_^–^ assimilation, which results in increased NUE and plant biomass. Additionally, we applied decreasing NO_3_^–^ treatments (from 8 to 6 and 4 mM NO_3_^–^) while maintaining the 6 mM Cl^–^ treatment (by replacing NO_3_^–^ by equivalent concentrations of SO_4_^2–^ + PO_4_^3–^ salts). Although leaf NO_3_^–^ contents were only slightly reduced after reducing 25 and 50%, the NO_3_^–^ concentration in the nutrient solution, total plant biomass strongly dropped up to 45% of the dry weight, coinciding with a slight reduction in NUE (Figures 3D–F). This is a consequence of the lower availability of NO_3_^–^ for the plant, causing a strong reduction in plant biomass.

### Effect of Cl^–^ on NUE Parameters in Different Plant Species

Considering these results, we hypothesized that a positive interaction between Cl^–^ nutrition and NUE is a widespread phenomenon in land plants. In order to answer this important question, herbaceous and woody plant species from different families with contrasting capacities to transport and accumulate Cl^–^ were tested in response to the 5 mM Cl^–^ treatment (Table 1). This study included several herbaceous and woody species of agricultural interest: leafy vegetables with strong Cl^–^-including capacity from the *Amaranthaceae* (chard) and the *Asteraceae* (lettuce) families; Cl^–^-including species from the *Solanaceae* family (tobacco and tomato); and two Cl^–^-excluding woody perennial species from the *Oleaceae* (olive) and the *Rutaceae* (the salt-tolerant citrus rootstock Cleopatra mandarin; Brumós et al., 2010).

**TABLE 1:** Effect of Cl^–^ nutrition on biomass, anion content and NUE parameters in different species of agronomic interest.

**Family**	**Species**	**N.T.**	**Total plant biomass (g DW)**	**Anion content and NUE parameters**					
				**Cl^–^ (mg g^–1^ DW)**	**NO_3_^–^ (mg g^–1^ DW)**	**TNC (mg g^–1^ DW)**	**NU_P_E (mg N g^–1^ root DW)**	**NU_T_E (g^2^ DW mg^–1^ N)**	**NUE (g DW mg^–1^ N)**
*Solanaceae*	Tomato	SP	34.20 ± 0.66	0.73 ± 0.02	4.10 ± 0.35	36.86 ± 0.54	707.9 ± 29.1	0.93 ± 0.02	105.05 ± 2.04
		CL	47.81 ± 0.79	32.33 ± 1.12	2.20 ± 0.41	31.85 ± 0.61	555.3 ± 31.2	1.51 ± 0.05	146.86 ± 2.42
		*P*-value	***	***	**	***	**	***	***
*Oleaceae*	Olive	SP	0.44 ± 0.03	1.67 ± 0.31	2.40 ± 0.09	27.70 ± 1.57	258.8 ± 14.6	0.016 ± 0.001	1.36 ± 0.10
		CL	0.40 ± 0.08	7.36 ± 0.79	1.71 ± 0.20	28.06 ± 1.28	257.1 ± 11.7	0.014 ± 0.003	1.24 ± 0.24
		*P*-value	**ns**	***	*	**ns**	**ns**	**ns**	**ns**
*Rutaceae*	Mandarin	SP	9.68 ± 0.13	1.03 ± 0.09	3.46 ± 0.52	26.07 ± 1.04	444.7 ± 14.3	0.37 ± 0.02	28.67 ± 0.33
		CL	11.02 ± 0.28	18.23 ± 0.36	2.11 ± 0.11	23.52 ± 0.48	452.6 ± 2.88	0.47 ± 0.01	32.29 ± 0.59
		*P*-value	**	***	*	*	**ns**	**	*
*Asteraceae*	Lettuce	SP	18.42 ± 0.91	16.47 ± 2.02	9.04 ± 0.20	23.94 ± 1.02	221.4 ± 9.47	0.78 ± 0.07	56.58 ± 2.80
		CL	27.95 ± 3.19	76.71 ± 2.13	7.56 ± 0.50	23.39 ± 1.48	175.9 ± 11.1	1.20 ± 0.14	85.86 ± 9.79
		*P*-value	*	***	*	**ns**	*	*	*
*Amaranthaceae*	Spinach	SP	7.23 ± 0.36	12.29 ± 1.07	4.79 ± 0.26	26.79 ± 1.39	247.7 ± 12.8	0.27 ± 0.02	45.25 ± 1.21
		CL	9.07 ± 0.37	80.86 ± 4.14	4.45 ± 0.05	25.22 ± 0.68	189.6 ± 5.14	0.36 ± 0.02	55.13 ± 2.97
		*P*-value	*	***	**ns**	**ns**	*	*	*
	Chard	SP	14.73 ± 0.39	10.82 ± 0.54	7.41 ± 0.34	21.48 ± 0.50	198.6 ± 4.60	0.69 ± 0.01	56.58 ± 2.80
		CL	17.95 ± 0.97	107.1 ± 3.35	5.57 ± 0.26	18.99 ± 0.59	142.8 ± 4.40	0.95 ± 0.07	85.86 ± 9.79
		*P*-value	*	***	**	*	***	*	*

Nutritional Treatment (N.T.) consisted of a basal nutrient solution supplemented with 5 mM Cl^–^ (CL) or the SO_4_^2–^ + PO_4_^3–^ (SP) salt mixture containing the same cationic balance in all treatments. Tomato (Solanum lycopersicum L.); Olive (Olea europaea L. ssp. europaea); Mandarin (Citrus reshni Hort. ex Tan.); Lettuce (Lactuca sativa L.); Spinach (Spinacia oleracea L.), and Chard (Beta vulgaris L. ssp. vulgaris convar. cicla). TNC (Total Nitrogen Content); NU_P_E (Nitrogen-Uptake Efficiency); NU_T_E (Nitrogen-Utilization Efficiency); NUE (Nitrogen-Use Efficiency). Mean values ± SE, n = 4–6. Levels of significance: P > 0.05 (“ns,” not significant differences);*P ≤ 0.05. **P ≤ 0.01. ***P ≤ 0.001. “Homogeneous group” statistics was calculated through ANOVA test. DW, dry weight.

**FIGURE 4:**
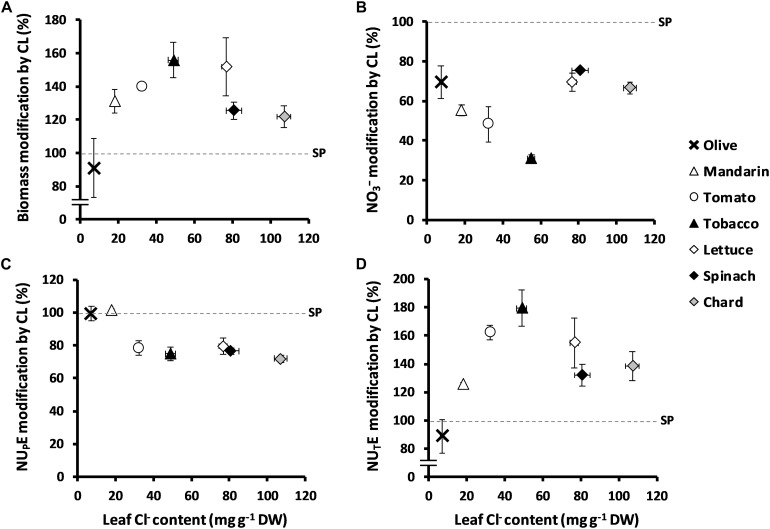
Effect of Cl^–^ nutrition on plant growth, NO_3_^–^ content, N uptake efficiency (NU_P_E), and N utilization efficiency (NU_T_E) in several species of agronomic interest. Plants were treated with two nutritional treatments: 5 mM Cl^–^ salts (CL) and a mixture of SO_4_^2–^ + PO_4_^3–^ salts (SP) containing the same cationic balance as in the CL treatment. Ratios of total biomass **(A)**, NO_3_^–^ content expressed as mg kg^–1^ of fresh weight **(B)**, NU_P_E **(C)**, and NU_T_E **(D)** are presented considering the % of CL in relation to SP treatment and in contrast to leaf anion content in several species. Olive (*Olea europaea* L. ssp. *europaea*; bold cross), mandarin (*Citrus reshni* Hort. ex Tan; open triangles), tomato (*Solanum lycopersicum* L.; open circles), tobacco (*Nicotiana tabacum* L.; filled triangles), lettuce (*Lactuca sativa* L.; open diamonds); spinach (*Spinacia oleracea* L.; filled diamonds), and chard (*Beta vulgaris* L. ssp. *vulgaris*; gray-colored diamonds); mean values ± SE, *n* = 6.

When treated with 5 mM Cl^–^, the Cl^–^-excluding species *O. europaea* and Cleopatra mandarin accumulated 7.36 and 18.23 mg Cl^–^ g^–1^ DW in leaf tissues, respectively; the Cl^–^-including tomato and tobacco plants accumulated 32.33 and 55.10 mg Cl^–^ g^–1^ DW in leaf tissues, respectively; and the strong Cl^–^-including leafy vegetables lettuce, spinach, and chard accumulated 76.71, 80.86, and 107.12 mg Cl^–^ g^–1^ DW, respectively. It is noteworthy that Cl^–^ improved biomass and NU_T_E in all the species tested (Figure 4), with the exception of olive, which was the species with the lowest Cl^–^ accumulation ability (Table 1). Thus, Cl^–^ stimulated plant biomass (Figure 4A), reduced leaf NO_3_^–^ content (Figure 4B and Supplementary Table S4) and NU_P_E (Figure 4C), and stimulated NU_T_E (Figure 4D). These responses showed a clear correlation with the content of Cl^–^ accumulated in the leaves of the different plant species, up to a value of ∼50 mg Cl^–^ g^–1^ DW in tobacco leaves. Species accumulating higher Cl^–^ contents showed a saturation response (Figure 4).

It is worth mentioning that, as previously described in tobacco plants (Franco-Navarro et al., 2016), Cl^–^ nutrition significantly increased water content of all the tested plant species except for the Cl^–^ excluders olive and Cleopatra mandarin (Supplementary Table S4). Notably, NO_3_^–^ content significantly decreased by the application of Cl^–^ in all species tested (Figure 4B and Supplementary Table S4). Regarding TNC, we observed that, in comparison to the SP treatment, the Cl^–^ treatment did not induce significant changes in olive, lettuce, and spinach (as in tobacco plants; Figure 1D), whereas a slight decrease was found in other species like tomato, Cleopatra mandarin, and chard (Table 1). Interestingly, NU_P_E was unaffected in the poor Cl^–^ including species (olive and Cleopatra mandarin), whereas it was moderately reduced (∼20%) in the Cl^–^-including species (Figure 4C). Thus, the increase in leaf Cl^–^ accumulation showed positive correlations with biomass and NU_T_E among the species (Figures 4A,D). These results indicate that the beneficial effect of Cl^–^ as a macronutrient on plant growth and NUE is a highly relevant phenomenon that could be extended to cultivated plants.

## Discussion

NO_3_^–^, an essential source of N, and Cl^–^, an important osmoregulatory molecule and beneficial macronutrient, are the most abundant inorganic anions in plants, and both must be coordinately incorporated during the active growth of plants (Cubero-Font et al., 2016; Colmenero-Flores et al., 2019). Both anions play important roles in charge balance and turgor regulation, showing strong dynamic interactions in land plants (Wege et al., 2017; Geilfus, 2018; Colmenero-Flores et al., 2019). Since NO_3_^–^ and Cl^–^ also present similar physical properties in solution, they share ion transport mechanisms with uncertain selectivities for both anions. NO_3_^–^, as a source of the essential macronutrient N, is assimilated during anabolic metabolism, while Cl^–^, which is not metabolized, becomes accumulated in plant tissues. Interaction between NO_3_^–^ and Cl^–^ has been traditionally understood as antagonistic. For instance, a high tissue content of Cl^–^ is believed to reduce the content of NO_3_^–^ and *vice versa* (Xu et al., 2000; Umar and Iqbal, 2007). The presence of external NO_3_^–^ has been shown to inhibit root Cl^–^ uptake (Glass and Siddiqi, 1985; Iglesias et al., 2004), and on the contrary, high Cl^–^ content reduces NO_3_^–^ accumulation in plants, suggesting that common transport mechanisms could facilitate the influx of both anions (Xu et al., 2000; Teakle and Tyerman, 2010). This antagonism between NO_3_^–^ and Cl^–^ has been widely reported for many crops, pointing to a clear detrimental effect of Cl^–^ on NO_3_^–^ nutrition (transport, accumulation, and/or assimilation; Buwalda and Smith, 1991; Cerezo et al., 1997; Xu et al., 2000). As a result, Cl^–^ is considered harmful to crop productivity, to the extent that its presence in some types of plant fertilizers is considered as a negative indicator of their quality (EU Regulation 2019/1009). However, Cl^–^ has been recently defined as a beneficial macronutrient that improves plant development, water relations, CO_2_ assimilation, and water-use efficiency when supplemented at concentrations higher than those necessary to satisfy micronutrient requirements but insufficient to cause toxicity (e.g., in the beneficial range of 1–5 mM Cl^–^; Colmenero-Flores et al., 2019; Franco-Navarro et al., 2019). The fact that Cl^–^ specifically promotes plant biomass due to these beneficial effects (Franco-Navarro et al., 2016) is difficult to reconcile with a detrimental effect on NO_3_^–^ nutrition.

Consistent with our previous findings (Franco-Navarro et al., 2016, 2019), tobacco plants accumulated Cl^–^ at levels that are typical of a macronutrient, stimulating plant growth when applied at concentrations above 1 mM Cl^–^ (Figures 1A, 2A). Furthermore, although leaf Cl^–^ content was ∼120 times lower in SP and N plants in comparison to CL, it remained over the critical threshold of Cl^–^ deficiency reported for non-halophytic plants (<0.2 mg g^–1^ shoot DW; Flowers, 1988; Xu et al., 2000; White and Broadley, 2001; Franco-Navarro et al., 2016), which ruled out the occurrence of Cl^–^ deficiency in SP and N treatments. Moreover, the higher growth of N plants (Figure 1A) confirmed this point and strengthens the well-known fact that NO_3_^–^ has a strong impact on plant growth and development (Glass, 2003; Hawkesford et al., 2012; Wang et al., 2012; Krapp et al., 2014; Guan, 2017).

NUE is an important crop trait described as a useful tool to improve agricultural systems (Fageria et al., 2008). This work clearly states that, contrary to what was previously believed, Cl^–^ improves NUE in plants, at least when NO_3_^–^ is used as the sole N source. The decline in leaf biomass has been directly correlated to N deficiency particularly in tobacco plants (Balachandran et al., 1997), since this crop requires high quantities of NO_3_^–^ for maximum vegetative yield (Ruiz et al., 2006). Considering that N is not only an essential nutrient for optimal crop yield but also an environmental concern, adequate management of N fertilization regimes to enhance NUE remains critical for crop breeding. Our results confirm that Cl^–^ significantly increases NUE, not only in tobacco plants (Figures 1, 2) but also in different crop species (Figure 4), when accumulated at macronutrient levels. NUE improvement in tobacco plants was a consequence of more efficient use of the NO_3_^–^ taken up by the plant (NU_T_E; Figures 2D, 3C), meaning that Cl^–^ improves NO_3_^–^ assimilation, as observed in other crop species (Figure 4D). A significant positive correlation (*r*^2^ = 0.995) between leaf Cl^–^ content and NU_T_E was established in tobacco plants (Figure 2F). Interestingly, this positive correlation was also observed in different plant species with contrasting abilities to accumulate Cl^–^ (Figure 4D). Thus, NU_T_E gain by Cl^–^ application was minimal in Cl^–^ excluding species (0–22% NU_T_E increment in olive and the citrus rootstock Cleopatra mandarin plants, respectively) and maximal in Cl^–^ including ones (60–80% NU_T_E increment in tomato and tobacco plants, respectively), indicating a positive ecophysiological correlation between leaf Cl^–^ accumulation and NU_T_E. However, this correlation was lost in strong Cl^–^ including vegetables (30–55% NU_T_E increment in the large-leaved spinach, chard, and lettuce plants), suggesting the occurrence of a saturable response, possibly as a consequence of excessive Cl^–^ accumulation. This positive correlation between Cl^–^ content and NU_T_E can be a selection criterion to identify new cultivars or genotypes obtained from breeding programs, with potentially improved NUE capacity. Thus, genotypes that, in the presence of 5 mM Cl^–^, show leaf Cl^–^ contents between 20 and 50 mg g^–1^ DW (Table 1), within the positive linear response range shown in Figure 4D, could be good candidates not only for improved NUE but also for higher efficiency in the use of water and CO_2_ (Colmenero-Flores et al., 2019).

These results were obtained comparing SP and CL treatments, both containing the same NO_3_^–^ concentration (5 mM NO_3_^–^). However, NU_T_E stimulation by Cl^–^ was much higher when the CL treatment was compared with the N treatment (10.25 mM NO_3_^–^). The increase in NU_T_E in CL vs. N tobacco plants was ∼250% (Figure 1F), suggesting that NO_3_^–^ fertilization in the field can be efficiently regulated if optimal supplies of NO_3_^–^/Cl^–^ ratios are used. Thus, increasing the Cl^–^/NO_3_^–^ ratio showed two positive effects on plants: reduction in leaf NO_3_^–^ content (Figure 3A) while at the same time increasing plant biomass (Figure 3B). Different studies have proposed a negative effect of Cl^–^ on NO_3_^–^ uptake and accumulation (Siddiqi et al., 1990; Cerezo et al., 1997; Li et al., 2017), which is supposed to reduce NUE. Nevertheless, our results ruled out the possibility that Cl^–^ impairs N use because the CL treatment increased plant biomass (Figure 3B), while the effective reduction in NO_3_^–^ in the nutrient solution produced a strong reduction in plant biomass (Figure 3E). This clearly indicates that the loss of leaf NO_3_^–^ content through Cl^–^ application is not a consequence of lower root NO_3_^–^ uptake (e.g., lower NO_3_^–^ availability as a consequence of Cl^–^ antagonism; Figure 2C) but of a greater NO_3_^–^ assimilation capacity. The NO_3_^–^ vs. Cl^–^ antagonism must be understood in terms of the selectivity of anion transporters. Given the great relevance of N for plant nutrition, plants prioritize NO_3_^–^ uptake over Cl^–^ uptake when NO_3_^–^ is available in the soil. This means that active transport mechanisms are normally more selective for NO_3_^–^ than for Cl^–^ (Glass and Siddiqi, 1985; Wege et al., 2017; Wen et al., 2017). Consequently, increasing the NO_3_^–^ concentration in the nutrient solution reduces Cl^–^ content in plants (Glass and Siddiqi, 1985; Iglesias et al., 2004). However, the opposite situation is not necessarily true. Although widely reported (Xu et al., 2000; and references therein), Cl^–^ application in the low millimolar range should not impair NO_3_^–^ uptake given the high selectivity for NO_3_^–^ over Cl^–^. The total N content of plants does not decrease in response to Cl^–^ application (Figure 1D; Ourry et al., 1992; Liu and Shelp, 1996; Inal et al., 1998). However, in Figure 4C, a moderate reduction in NU_P_E can be observed in different plant species in response to Cl^–^ application. Rather than an effective reduction in NO_3_^–^ uptake transport through transmembrane transporters at the soil–root interface, NU_P_E reduction can be a consequence of the calculation procedure. The NU_P_E formula computes the NO_3_^–^ content in plant tissues, which is lower in plants treated with Cl^–^ because NO_3_^–^ is more efficiently assimilated, as also proposed by Liu and Shelp (1996). It is very likely, however, that under salinity stress conditions, Cl^–^ antagonizes NO_3_^–^ influx in plant cells, significantly reducing root NO_3_^–^ uptake (Cerezo et al., 1997; Li et al., 2017).

Therefore, our results strongly support the previously suggested role of Cl^–^ as preferred plant osmoregulatory molecule in plants (Flowers, 1988; Franco-Navarro et al., 2016; Colmenero-Flores et al., 2019). Thus, we propose that, on the one hand, Cl^–^ is preferably compartmentalized in the vacuole. On the other hand, NO_3_^–^, an essential N source for land plants, is preferentially assimilated, which is not possible when this molecule is sequestered in the vacuole to carrry out an osmotic function. Only when Cl^–^ is not sufficiently available in the soil, or as a result of excessive NO_3_^–^ availability, NO_3_^–^ could be preferentially compartmentalized (Siddiqi et al., 1991; Radcliffe et al., 2005). Therefore, macronutrient accumulation of Cl^–^ reduces NO_3_^–^ compartmentalization in the vacuole, facilitating its assimilation, which increases NUE and plant biomass. Under the same premise, Cl^–^ should also play an adaptive role to improve plant growth under conditions of low N availability, which is also explained in terms of differential transport selectivity. When little NO_3_^–^ is available, root Cl^–^ uptake through active anion transporters is less inhibited (Wen et al., 2017), increasing cell Cl^–^ content and replacing NO_3_^–^ in the vacuole, which facilitates NO_3_^–^ assimilation and NUE. A clear demonstration that the relationship between Cl^–^ and NO_3_^–^ homeostasis in higher plants is not limited to an antagonistic interaction has been recently shown by Cubero-Font et al. (2016). This work describes a molecular mechanism that determines the rate of NO_3_^–^/Cl^–^ accumulation in aerial organs of *Arabidopsis thaliana* based on the Cl^–^ conductance of the AtSLAH3 channel, which is, in turn, regulated by environmental cues.

Agronomic and scientific communities have traditionally believed that little amounts of Cl^–^ are required to achieve suitable crop yields (Geilfus, 2018). Nevertheless, some studies have shown that the application of Cl^–^-enriched fertilizers to the soil increases the vegetative yield in different crops (Christensen et al., 1981; Timm et al., 1986; Inal et al., 1998; Xu et al., 2000). However, it was not clear to what extent plant yield improvement was due to the accompanying cations or whether other anions could replace Cl^–^ in the reported growth-promoting effects. In accordance with the recently revealed functions of Cl^–^ as a beneficial macronutrient (Franco-Navarro et al., 2016; Colmenero-Flores et al., 2019), it has been proven that a number of physiological perturbations impairing the growth and yield of durum wheat under field conditions are specifically due to soil Cl^–^ deficiency (Schwenke et al., 2015). Hence, we investigated how crops could benefit from certain levels of Cl^–^ fertilization. In the herbaceous species studied (i.e., tomato, lettuce, spinach, and chard), the 5 mM Cl^–^ treatment determined plant biomass gains following the leaf Cl^–^ content within the beneficial macronutrient range (40–110 mg g^–1^ DW; Colmenero-Flores et al., 2019; Figure 3B). These Cl^–^ content values are up to an order of magnitude above what was classically considered toxic concentrations in plants (Xu et al., 2000), largely dismantling this view of Cl^–^ as detrimental to agriculture (Colmenero-Flores et al., 2019).

Given the high NO_3_^–^ content in fertilizers and its often abusive use in agriculture, NO_3_^–^ can be excessively accumulated in leaves of most horticultural crops, resulting in food safety problems (e.g., methemoglobinemia and cancer) because of its transformation into nitrites and nitrosamines (Colla et al., 2018). This is particularly harmful in leafy vegetables, for which the European Commission has developed severe regulations (1881/2006 and 1258/2011) to reduce the excessive dietary intake of NO_3_^–^, especially that of vulnerable people such as infants, the elderly, and vegetarians. As previously stressed, increasing the Cl^–^/NO_3_^–^ ratios reduced the leaf NO_3_^–^ content (Figure 3A) without impairing, or even increasing, plant biomass (Figure 3B). In our study, the NO_3_^–^ content in leafy species (lettuce, spinach, and chard) treated with SP ranged between 577 and 1,035 mg NO_3_^–^ kg^–1^ FW (Supplementary Table S4), proving to be much lower than the maximum permitted levels, which are set at 3,500 and 2,500 mg NO_3_^–^ kg^–1^ FW in spinach and iceberg lettuce, respectively. It should be noted, however, that the SP treatment contains 5 mM NO_3_^–^, probably well below the levels applied in the field by farmers. Chloride reduced about 25–70% the NO_3_^–^ content in the plant species assayed (compared to SP plants; Figure 4B). These results are in accordance with those reported by Urrestarazu et al. (1998) in lettuce, Inal et al. (1998) in carrot, and Borgognone et al. (2016) in cardoon. Therefore, Cl^–^ nutrition is expected to considerably improve the nutritional quality of vegetables and brings to light the important benefits of using Cl^–^-enriched fertilizers in human health. Interestingly, Cl^–^-treated tobacco plants showed the strongest decrease in NO_3_^–^ content (∼70% compared to SP plants; Figure 4B). Considering that NO_3_^–^ is the main inducer of nitrogen oxides and nitrosamines in flue-cured tobacco during smoking (Hoffmann and Hecht, 1985), Cl^–^ nutrition could also help to reduce the nitrosamine levels in cigarettes, improving the quality of this crop.

## Conclusion

We provide for the first time a direct demonstration which shows that Cl^–^, contrary to impairing NO_3_^–^ nutrition, facilitates NO_3_^–^ utilization and improves NUE in plants. This is largely due to Cl^–^ improvement of NU_T_E, having a little or moderate effect on NU_P_E when NO_3_^–^ is used as the sole N source in the nutrient solution. Clear positive correlations between leaf Cl^–^ content vs. NU_T_E or vs. plant growth have been established at both intra- and interspecies levels: in tobacco plants treated with growing Cl^–^ concentrations and comparing different species with contrasting abilities to accumulate Cl^–^. Our results strongly suggest that macronutrient Cl^–^ nutrition reduces NO_3_^–^ sequestration in plant leaf tissues (e.g., vacuolar compartmentalization), making this valuable N source available for assimilation and biosynthesis of organic N. Our results give light to a brand-new interpretation of Cl^–^ properties as a beneficial macronutrient for higher plants that promote more efficient use of water, carbon, and nitrogen, becoming a potential resource to improve agricultural production and quality, reducing NO_3_^–^ inputs in the field and unhealthy leaf NO_3_^–^ content in vegetables.

## Data Availability Statement

The datasets generated for this study are available on request to the corresponding author.

## Author Contributions

JF-N performed the experiments, analyzed the data, and participated in the writing of the manuscript. PP-T and PD-R participated in the experiments. RÁ participated in the conception of research plans. MR and JC-F conceived research plans, supervised the experiments, and wrote the manuscript.

## Conflict of Interest

The authors declare that the research was conducted in the absence of any commercial or financial relationships that could be construed as a potential conflict of interest.
