# Supplementary material for: Chloride Improves Nitrate Utilization and NUE in Plants
Source: Front Plant Sci. 2020 May 26;11:442. doi: 10.3389/fpls.2020.00442 (PMC7264407; doi:10.3389/fpls.2020.00442)
Supplement: Supplementary file 1 [file Data_Sheet_1.doc]

| **Supplementary Table 1**. Relation of nutritional treatments. | | | | | | | | |
| --- | --- | --- | --- | --- | --- | --- | --- | --- |
| **Treatments** | **Ion concentration in the irrigation treatment (mM)** | | | | | | | |
|  | **Cl**^−^ | **NO_3_**^−^ | **SO_4_^2^**^−^ | **PO_4_^3^**^−^ | **SO_4_^2^**^−^ **+ PO_4_^3^**^−^ | **K^+^** | **Ca^2+^** | **Mg^2+^** |
| **SP 5 mM** | 0.075 | 5.25 | 3.015 | 2.048 | 5.063 | 4.62 | 2.63 | 1.63 |
| **N 5 mM** | 0.075 | 10.25 | 1.140 | 0.798 | 1.938 | 4.62 | 2.63 | 1.63 |
| **Cl 5 mM** | 5.075 | 5.25 | 1.140 | 0.798 | 1.938 | 4.62 | 2.63 | 1.63 |
| **SP 0.15 mM** | 0.075 | 5.25 | 1.190 | 0.840 | 2.030 | 2.19 | 2.02 | 1.02 |
| **SP 0.30 mM** | 0.075 | 5.25 | 1.250 | 0.870 | 2.120 | 2.27 | 2.04 | 1.04 |
| **SP 1 mM** | 0.075 | 5.25 | 1.510 | 1.050 | 2.560 | 2.62 | 2.13 | 1.13 |
| **SP 2.5 mM** | 0.075 | 5.25 | 2.080 | 1.420 | 3.500 | 3.37 | 2.31 | 1.31 |
| **SP 5 mM** | 0.075 | 5.25 | 3.015 | 2.048 | 5.063 | 4.62 | 2.63 | 1.63 |
| **CL 0.15 mM** | 0.151 | 5.25 | 1.140 | 0.798 | 1.938 | 2.19 | 2.02 | 1.02 |
| **CL 0.30 mM** | 0.301 | 5.25 | 1.140 | 0.798 | 1.938 | 2.27 | 2.04 | 1.04 |
| **CL 1 mM** | 1.075 | 5.25 | 1.140 | 0.798 | 1.938 | 2.62 | 2.13 | 1.13 |
| **CL 2.5 mM** | 2.575 | 5.25 | 1.140 | 0.798 | 1.938 | 3.37 | 2.31 | 1.31 |
| **CL 5 mM** | 5.075 | 5.25 | 1.140 | 0.798 | 1.938 | 4.62 | 2.63 | 1.63 |
| In most experiments the nutritional treatments were: the basal nutrient solution supplemented with 5 mM Cl^−^ (CL), 5 mM NO_3_^−^ (N) or the SO_4_^2−^ + PO_4_^3−^ (SP) salt mixture containing the same cationic balance as in the CL and N supplements. In the experiment of Figure 2, basal solution (0.075 mM  Cl^−^) was supplemented with 0.15, 0.3, 1, 2.5 or 5 mM Cl^−^ (CL), and alternatively the same increasing concentrations with the SO_4_^2−^ + PO_4_^3−^ (SP) salt mixture containing the same cationic balance as in the CL supplements. Red text indicates anions, and blue text indicates cations. Colored boxes lines denote the concentration of the main anion(s) in each treatment. | | | | | | | | |

| **Supplementary Table 2**. Relation of nutritional treatments. | | | | | | |
| --- | --- | --- | --- | --- | --- | --- |
| **Treatment** | **Ion concentration in the irrigation treatment (mM)** | | | | | |
|  | **Cl**^−^ | **NO_3_**^−^ | **SO_4_^2^**^−^ **+ PO_4_^3^**^−^ | **K^+^** | **Ca^2+^** | **Mg^2+^** |
| **8 : 0.075** | 0.075 | 8.0 | 8.0 | 9.2 | 2.6 | 2.6 |
| **8 : 0.5** | 0.575 | 8.0 | 7.5 | 9.2 | 2.6 | 2.6 |
| **8 : 2** | 2.075 | 8.0 | 6.0 | 9.0 | 2.5 | 2.5 |
| **8 : 4** | 4.075 | 8.0 | 4.0 | 8.8 | 2.3 | 2.3 |
| **8 : 6** | 6.075 | 8.0 | 2.0 | 8.4 | 2.2 | 2.2 |
| **6 : 4** | 6.075 | 6.0 | 4.0 | 8.6 | 2.3 | 2.3 |
| **4 : 6** | 6.075 | 4.0 | 6.0 | 9.0 | 2.4 | 2.4 |
| In the experiment of Figure 3, treatments consisted of the application of: (i; ↑Cl^−^ : ↓SO_4_^2−^ + PO_4_^3−^) increasing concentration of Cl^−^ (from 0.075 to 6 mM), decreasing the concentration of SO_4_^2−^ + PO_4_^3−^ (from 8 to 2 mM), while keeping constant the concentration of NO_3_^−^ (8 mM); and (ii; ↓NO_3_^−^ : ↑SO_4_^2−^ + PO_4_^3−^) keeping constant the concentration of Cl^-^ (6 mM), increasing the concentration of SO_4_^2−^ + PO_4_^3−^ (from 2 to 6 mM), and decreasing the concentration of NO_3_^−^ (from 8 to 4 mM). Red text indicates anions, and blue text indicates cations. | | | | | | |

| **Supplementary Table 3.** Leaf ion content in tobacco plants subjected to different treatments. | | | | | | | |
| --- | --- | --- | --- | --- | --- | --- | --- |
| **Treatment** | **Ion content (mg g^-1^ DW)** | | | | | | |
|  | **Cl**^−^ | **NO_3_**^−^ | **PO_4_^3^**^−^ | **SO_4_^2^**^−^ | **K^+^** | **Ca^2+^** | **Mg^2+^** |
| **SP** | 0.42 ± 0.04 b | 2.24 ± 0.43 b | 13.92 ± 0.18 a | 35.01 ±2.40 a | 48.41 ± 2.32 | 14.25 ± 1.34 | 8.16 ± 1.11 |
| **N** | 0.56 ± 0.07 b | 10.24 ± 0.58 a | 10.37 ± 0.19 b | 11.91 ± 0.96 b | 49.24 ± 2.36 | 15.63 ± 0.63 | 7.41 ± 0.80 |
| **CL** | 55.10 ± 1.05 a | 1.03 ± 0.07 c | 11.07 ± 0.12 b | 12.31 ± 0.15 b | 49.56 ± 1.84 | 15.29 ± 2.16 | 7.69 ± 0.77 |
| ***P*-value** | ******* | ******* | ******* | ******* | **ns** | **ns** | **ns** |
| Treatments consisted of the basal nutrient solution supplemented with 5 mM Cl^−^ (CL), 5 mM NO_3_^−^ (N) or the SO_4_^2−^ + PO_4_^3−^ (SP) salt mixture containing the same cationic balance as in the CL and N supplements. Mean values ± SE, *n* = 6. Levels of significance: *P* > 0.05 (ns, not significant differences); *P* ≤ 0.001 (***). “Homogeneous group” statistics was calculated through ANOVA. | | | | | | | |

| **Supplementary Table 4.** Effect of Cl^−^ nutrition on water and NO_3_^−^ content in different species of agronomic interest. | | | | |
| --- | --- | --- | --- | --- |
|  |  |  | **Water**  **(%)** | **NO_3_**^−^ **(mg Kg^-1^ FW)** |
| **SOLANACEAE** | **Tobacco** | **SP** | 89.3 ± 0.62 | 240.6 ± 46.2 |
|  |  | **CL** | 92.8 ± 0.33 | 74.7 ± 5.08 |
|  |  | ***P*-value** | ***** | ******* |
|  | **Tomato** | **SP** | 90.5 ± 0.20 | 391.1 ± 33.4 |
|  |  | **CL** | 91.4 ± 0.24 | 188.9 ± 35.2 |
|  |  | ***P*-value** | ***** | ******* |
| **OLEACEAE** | **Olive** | **SP** | 72.1 ± 0.86 | 670.3 ± 25.1 |
|  |  | **CL** | 72.8 ± 0.40 | 465.6 ± 54.4 |
|  |  | ***P*-value** | ns | ****** |
| **RUTACEAE** | **Mandarin** | **SP** | 70.3 ± 0.02 | 1027.3 ± 154.4 |
|  |  | **CL** | 73.1 ± 1.74 | 567.4 ± 29.6 |
|  |  | ***P*-value** | ns | ******* |
| **ASTERACEAE** | **Lettuce** | **SP** | 90.4 ± 0.91 | 870.6 ± 19.3 |
|  |  | **CL** | 92.5 ± 0.33 | 604.8 ± 40.0 |
|  |  | ***P*-value** | ***** | ******* |
| **AMARANTHACEAE** | **Spinach** | **SP** | 88.0 ± 0.33 | 577.2 ± 31.3 |
|  |  | **CL** | 90.2 ± 0.33 | 435.6 ± 4.89 |
|  |  | ***P*-value** | ****** | ****** |
|  | **Chard** | **SP** | 86.0 ± 0.27 | 1035.9 ± 47.5 |
|  |  | **CL** | 87.6 ± 0.40 | 689.6 ± 32.2 |
|  |  | ***P*-value** | ***** | ******* |
| Nutritional Treatment (N.T.) consisted of a basal nutrient solution supplemented with 5 mM Cl^−^ (CL) or the SO_4_^2−^ + PO_4_^3−^ (SP) salt mixture containing the same cationic balance in all treatments. Plants of several species were treated with two nutritional treatments: 5 mM chloride salts (CL) and a mixture of sulphate + phosphate salts (SP) containing the same cationic balance as in the CL treatment. NO_3_^−^ content ratio is presented considering the % of CL in relation to SP-treatment and in contrast to leaf anion content in several species. Several plant species were used: Olive (*Olea europaea* L. ssp. *europaea*); Mandarin (*Citrus reshni* Hort. ex Tan); Tomato (*Solanum lycopersicum* L.); Lettuce (*Lactuca sativa* L.); Spinach (*Spinacia oleracea* L.); Chard (*Beta vulgaris* L. ssp. *vulgaris*); Mean values ± SE, *n* = 6. Levels of significance: *P* > 0.05 (‘ns’, not significant differences); *P* ≤ 0.05 (*). *P* ≤ 0.01 (**). *P* ≤ 0.001 (***). “Homogeneous group” statistics was calculated through ANOVA test. FW, fresh weight. | | | | |
